# Baseline results of the first malaria indicator survey in Iran at household level

**DOI:** 10.1186/1475-2875-10-277

**Published:** 2011-09-22

**Authors:** Mahdi Mohammadi, Alireza Ansari-Moghaddam, Ahmad Raiesi, Fatemeh Rakhshani, Fatemeh Nikpour, Aliakbar Haghdost, Mansoor Ranjbar, Rahim Taghizadeh-Asl, Mohammad Sakeni, Reza Safari, Mehdi Saffari

**Affiliations:** 1 Health Promotion Research Center, Zahedan University of Medical Sciences, Zahedan, Iran; 2 Center for Disease Control & Prevention, Ministry of Health and Medical Education, Tehran, Iran; 3 Physiology Research Center, Kerman University of Medical Sciences, Kerman, Iran; 4 United Nation Development Program, Tehran, Iran; 5 Province Health Center, Zahedan University of Medical Sciences, Zahedan, Iran; 6 Province Health Center, Hormozgan University of Medical Sciences, Bandar Abbas, Iran; 7 Province Health Center, Kerman University of Medical Sciences, Kerman, Iran

## Abstract

**Background:**

Malaria is one of the leading causes of sickness and death in the developing world, causing more than a million deaths and around 250 million new cases annually worldwide. The aim of this comprehensive survey was to provide information on malaria indicators at household level in high-risk malaria areas in Iran.

**Methods:**

In a cluster randomized cross-sectional survey data were collected from 5,456 households in both rural and urban areas of 20 malaria-affected districts of Iran. All the fieldwork was done by trained interviewers and a validated questionnaire. The questionnaire comprised baseline characteristics of the study population, the knowledge of people about different aspects of malaria (such as clinical symptoms, transmission and prevention) and their practice to prevent illness (such as using mosquito nets, spraying houses). The data were analysed and descriptive statistics (i.e. frequencies, percentages) were used to summarize the results.

**Results:**

The results of this survey showed that 20% (95% CI: 17.36 - 22.24) of households owned at least one mosquito net, whether treated or untreated. Consequently, the use of mosquito nets was considerably low among both children under age five [5.90% (95% CI: 5.14 - 6.66)] and pregnant women [5.70% (95% CI: 3.07 - 8.33)]. Moreover, less than 10% of households reported that the interior walls of their dwelling had been sprayed in the previous year [8.70% (95% CI: 6.09 - 11.31)]. Data also suggest that 63.8% of the participants recognized fever as a sign of malaria, 56.4% reported that mosquito bites cause malaria and about 35% of participants mentioned that the use of mosquito nets could prevent malaria.

**Conclusion:**

Findings from this study indicate that low access to treated nets along with low understanding of the role of nets in malaria prevention are the main barriers to utilization of bed nets. Therefore, the use of insecticide-treated mosquito nets should be encouraged through health education on the importance of the use along with increasing access to it.

## Background

Malaria is still one of the major health challenges, particularly in developing countries. According to the World Health Organization (WHO) report, half of the world's populations are currently in potential risk of contracting malaria [1]. Annually, approximately 250 million new cases of malaria are diagnosed, and about a million people die from malaria worldwide [1-3].

Sub-Saharan Africa constitutes a significant proportion of the global burden of malaria morbidity and mortality accounting for 85% of cases and 87% of deaths due to malaria in the world [1-3]. Nevertheless, approximately 60% of the populations of the Eastern Mediterranean Region are also at risk of malaria [3]. The Islamic Republic of Iran is one of the countries located in the Eastern Mediterranean Region with low malaria endemicity in some of its regions [3-6]. The burden of malaria in Iran has dropped considerably over the past three decades due to extensive malaria control programmes [4-6]. However, the south-eastern provinces of Iran (Sistan & Baluchestan, Hormozgan and the tropical part of Kerman) are still considered as malaria-endemic regions, accounting for around 95% of all malaria cases in the country [4-6].

Therefore, the Ministry of Health and Medical Education in Iran has focused its efforts on the Roll Back Malaria strategies in the control of malaria [7-9], including protecting 80% of children and pregnant women at risk for malaria with appropriate control methods, such as insecticide-treated nets, and, where appropriate, indoor residual spraying, early case detection and prompt correct treatment as well as accomplishment of health education and communication program to promote health-seeking behaviours and demands for services and products among family members.

However, to date no comprehensive malaria survey has been conducted to identify the coverage of these strategies in Iran. Additionally, the existing evidence about malaria indicators in the country, which comes mainly from routinely-collected data, is not sufficient to measure progress towards achieving national and regional goals and targets properly.

The present study was conducted to collect timely information on access, coverage and use of key malaria prevention and control programmes at the levels of household within 20 malaria-affected districts (targeted for Global Fund malaria project) in the three above-mentioned regions.

## Methods

### Study population

The south-eastern provinces of Iran including 60% of the total population of Sistan & Baluchestan, the whole population of Hormozgan and 30% of the total population of Kerman provinces are considered as malaria endemic regions in Iran. These malarious regions comprising of 20 urban and rural malaria-affected districts with a population of about three million people formed the study population distributed in Sistan & Baluchestan, Hormozgan and Kerman provinces with an approximate proportion of 40%, 40% and 20%, respectively. The proportions of population living in urban areas of the three provinces, however, were 26%, 36%, and 36% in Sistan & Baluchestan, Hormozgan and Kerman, respectively.

### Sample design

In a multistage random sampling, 125 clusters with an average size of 40 households were selected randomly from the study population. Firstly, the required sample size was divided based on the proportion of population in the three target provinces. Hence, there were 50 clusters in Sistan & Baluchestan, 50 clusters in Hormozgan and 25 clusters in Kerman province. Furthermore, since each province is, in turn, administratively subdivided into several districts and each district includes several rural and urban areas, the sample size of each province was divided based on the proportion of population in rural and urban areas. Accordingly and proportionately, 13, 18 and 9 clusters of required sample sizes were devoted to urban areas of Sistan & Baluchestan, Hormozgan and Kerman, respectively. The remaining clusters in each province were selected from rural areas.

Finally, for the purpose of this survey, all the health centers/units in the target districts in the three mentioned provinces were listed based on geographical regions and separately for urban and rural and then the populations were calculated cumulatively. At this stage, clusters and head-clusters (the first selected household as opening point for survey) were determined using systematic random sampling method. After that, trained personnel referred to the first household in every cluster and moved on their right side to cover the entire forty households in every cluster. Having used this approach, 5,456 households were surveyed.

### Survey questionnaire

After reviewing comparable questioners in other countries [10-14] and also available questionnaires in the website of WHO [15-17], the first draft of questionnaire was developed and shared with experts within the Ministry of Health. Based on feedback received, the pre-final questionnaire was developed and piloted to check its validities and reliability. Finally, a meeting was held with the participation of the general director of malaria in Iran, the coordinator and the representative of Global Fund, main investigators, and a group of knowledgeable malaria experts from all three provinces. In this meeting, the contents of the questionnaire was reviewed item by item and modified if necessary. Then, the questionnaire and its completion instructions were finalized for the survey.

The questionnaire comprised information about under five-year old children and pregnant women, also included questions about the knowledge of people about different aspect of malaria (such as clinical symptoms, transmission, and prevention) and their practice to prevent illness (like using mosquito nets, spraying houses) or the treatment of the disease.

### Training and field work

Interviewers were trained in a combination course of classroom training and practical experience to know how to perform their task perfectly. Additionally, each interviewer had given a detailed manual, which was designed in accordance with WHO recommendations [15-17], to clarify them how to do their work.

### Data management and analysis

Data were collected through direct referring of the trained interviewing teams to the selected households and filling in questionnaires. Once all the information was collected and cleaned, analysis was done and descriptive statistics (i.e. frequencies, percentages) were used to estimate coverage, use and access estimates. Point estimates and 95% confidence intervals were estimated for all malaria indicators and sample characteristics. Relevant parametric or non-parametric statistical test, such as pearson's Chi squared test and the homogeneity test of Chi squared were used to determine association or heterogeneity with a probability of committing a type 1 error (a) was set at 0.05.

### Evaluation the quality of project implementation

The quality of data collection was daily supervised and monitored by main investigators, provincial and district focal points during implementation of the project to ensure the quality and quantity of data collected by surveyors. Additionally, the quality of data entering was also monitored in two stages: in the primary stage, the data file and its manual was prepared by skilled experts in statistics. Then, data were entered by a team of trained data entry operators under the direct supervision of specialists in statistics and epidemiology. In the final stage, as data were analysed the information about every variable was controlled once more and if necessary was corrected by referring to the questionnaires.

### Ethical clearance

This study was approved by the research ethics committee of Zahedan University of Medical Sciences, Iran. Additionally, the aims of the project were discussed with all provincial health authorities and their written or verbal informed consent was received for cooperation in this case. Moreover, verbal, informed consent was obtained from the heads of households and each eligible individual before conducting the household questionnaires. In an effort to maintain confidentiality, the names of respondents and households were kept as strictly confidential information and were not to be used in the presentation of results. Collected personal data were given a code number and only to be accessed by the principal investigators or with permission from the principal investigator.

## Results

### Characteristics of study population

In the current study, a total of 5,456 households (2,057 households from Sistan and Balucehstan, 2,353 from Hormozgan and 1,046 from Kerman province) were surveyed in both rural and urban area of three target provinces of Iran.

### Household net ownership

Overall, one out of every five households owned at least one or more mosquito nets (Table 1). However, the percentage of households with at least one mosquito net was substantially higher in rural areas compared to the urban areas [25.2; 95% CI: 23.8 - 26.6%] vs. [5%; 95% CI: 3.90 - 6.10]. Additionally, there was a large variation among sampled provinces of Iran; 40.6% [95% CI: 38.5 - 42.8] of surveyed families in Sistan and Baluchestan(S&B) province owned at least one mosquito net of any type compared to less than 10% in the other two regions.

**Table 1:** Households with at least one and more than one mosquito net (treated or untreated)

	Having bed nest	
	
Province	Frequency (%)	95% CI
	
S&B	834 (40.6)	38.5 - 42.8
Hormozgan	109 (4.80)	3.89 - 5.63
Kerman	82 (9.80)	7.82 - 11.9
Residency		
Urban	75 (5.00)	3.90 - 6.10
Rural	921 (25.2)	23.8 - 26.6
**Total**	1025 (19.8)	17.4 - 22.2

### Utilization of mosquito nets

Overall, a tiny proportion of children and pregnant women (about 6%) used mosquito net the night preceding the survey (Table 2). However, usage of mosquito nets varied by residency and region. Rural children were more likely than urban children to sleep under a net [6.56 (95% CI: 5.62 - 7.49) versus 2.50 (95% CI: 1.48 - 3.41); P for heterogeneity = 0.001]. Likewise, pregnant women in urban areas were less likely than those in rural areas [(0 vs. 5.88% (95% CI: 2.65 - 9.11); P for heterogeneity = 0.001) to sleep under a mosquito net. Additionally, results by region showed differences, with proportions of children under 5 and pregnant women used a mosquito net the night before of survey varying from less than equal 2% in Kerman to about 10% in the S&B province.

**Table 2:** Utilization of mosquito nets by vulnerable groups the night before of survey

	Children under 5 yrs		Pregnant women	
	
Province	Frequency (%)	95% CI	Frequency (%)	95% CI
	
S&B	158 (9.70)	8.30 - 11.2	10 (6.90)	2.77 - 11.0
Hormozgan	46 (3.10)	2.21 - 3.97	7 (5.70)	1.61 - 9.86
Kerman	13 (2.21)	1.02 - 3.40	0.00	0.00
Residency				
Urban	24 (2.50)	1.84 - 3.41	0.00	0.00
Rural	176 (6.56)	5.62 - 7.49	12 (5.88)	2.65 - 9.11
**Total**	217 (5.90)	5.14 - 6.66	17 (5.70)	3.07 - 8.33

### Indoor residual spraying (IRS)

On the whole, less than 10% of all the surveyed households reported that their houses had been sprayed in the previous twelve months by the district health teams. Again, the results indicated large differences in the percentage of households/population reached by IRS in the year preceding the survey by residency (P = 0.001) and regions (P = 0.001) (Table 3).

**Table 3:** Frequency of households & people in target areas reached by IRS

	Households reached by IRS		People reached by IRS	
	
Province	Frequency (%)	95% CI	Frequency (%)	95% CI
	
S&B	274 (13.3)	11.9 - 14.8	1360 (13.3)	12.6 - 14.0
Hormozgan	173 (7.60)	6.50 - 8.70	918 (8.10)	7.61 - 8.62
Kerman	1 (0.10)	0.00 - 0.35	2 (0.04)	0.00 - 0.09
Residency				
Urban	8 (0.56)	0.17 - 0.91	35 (0.46)	0.31 - 0.61
Rural	414 (11.4)	10.3 - 12.4	2099 (11.2)	10.7 - 11.6
**Total**	448 (8.70)	6.09 - 11.3	2280 (8.60)	8.23 - 8.91

### General malaria knowledge

In general, 63.8% [95% CI: 62.2 - 65.4] of respondents recognized fever as a sign of malaria while only one-third of them cited shivering as a symptom of malaria (Table 4). Additionally, just above half of participants 56.4 [95% CI: 54.6 - 58.2] reported that mosquito bites cause malaria. Notably, 34.9% [95% CI: 32.7 - 37.1] of participants mentioned that use of mosquito nets could prevent malaria. Furthermore, about one-third of selected samples in target districts did not know symptoms, transmission route and appropriate prevention method of malaria. Data also suggests a slight variation by residency, but substantial discrepancy according to the region. Participants in highly malarious area of S&B were more likely to cite malaria symptoms, its transmission route and effective prevention method than those in Hormozgan or Kerman province.

**Table 4:** Respondents knowledge about malaria symptoms, transmission and prevention

Respondent's awareness	Residency		Province			Total
		
	Rural	Urban		Hormozgan	Kerman	
Fever as malaria symptom	62.1 (60.5 - 63.6)	67.4 (65.1 - 69.7)	81.4 (79.7 - 83.0)	48.1 (46.2 - 50.2)	63.6 (60.7 - 66.5)	63.8 (62.2 - 65.4)
Shivering as malaria symptom	36.2 (34.7 - 37.8)	35.2 (32.8 - 37.5)	46.6 (44.4 - 48.8)	23.4 (21.7 - 25.1)	42.5 (39.6 - 45.5)	35.9 (33.7 - 38.9)
Malaria transmit through mosquito sting	55.4 (53.8 - 57.0)	59.1 (56.7 - 61.6)	77.2 (75.4 - 79.0)	36.6 (34.7 - 38.6)	59.1 (56.1 - 62.1)	56.4 (54.6 - 58.2)
Using bed net as preventive measure	38.4 (36.8 - 39.9)	25.6 (23.4 - 27.7)	64.0 (61.9 - 66.0)	10.0 (8.71 - 11.2)	31.3 (28.5 - 34.1)	34.9 (32.7 - 37.1)

### Community approach to onset of fever and shivering

The survey also asked respondents about their reaction when one of family members is having fever or shivering. Overall, 86.6% [95% CI: 85.6% - 87.6%] of individuals preferred to be treated professionally by health staff with no evidence of heterogeneity between rural and urban areas (Table 5).

**Table 5:** Respondents reaction when having fever and shivering

Reaction	Residency		Province			Total
		
	Rural	Urban	S & B	Hormozgan	Kerman	
To be treated professionally	87.1 (86.0 - 88.2)	86.1 (84.4 - 87.8)	94.4 (93.4 - 95.4)	81.0 (79.3 - 82.6)	82.4 (79.8 - 85.0)	86.6 (85.6 - 87.6)
Others	9.60 (8.64 - 10.6)	11.8 (10.1 - 13.4)	1.30 (0.78 - 1.75)	18.3 (16.7 - 19.9)	11.0 (8.89 - 13.1)	10.3 (7.71 - 12.8)
Taking anti-temperature drug	3.80 (3.02 - 4.67)	4.00 (2.61 - 5.30)	0.60 (0.25 - 0.91)	0.00	12.0 (9.77 - 14.2)	3.90 (3.17 - 4.58)
Do not know	3.00 (2.41 - 3.50)	2.30 (1.52 - 3.03)	2.50 (1.81 - 3.15)	2.80 (2.16 - 3.53)	3.50 (2.23 - 4.71)	2.80 (0.11 - 5.49)
Taking traditional remedies	2.00 (1.45 - 2.45)	1.50 (0.91 - 2.16)	3.00 (2.23 - 3.70)	1.20 (0.79 - 1.70)	1.60 (0.72 - 2.40)	2.00 (0.00 - 4.72)

## Discussion

This first comprehensive assessment of the coverage of the key malaria indicators in Iran highlights several important areas that need to be addressed urgently by Ministry of Health and Medical Education to achieve malaria control targets set by RBM partnership as well as national strategic plan for malaria elimination.

The current study confirmed low access to mosquito net among surveyed households. Based on findings in this report, only one-fifth of households owned at least one bed net (treated or untreated). Accordingly, tiny proportion of children under five years of age (6%) and pregnant women (6%) slept under any net the night before the survey. Net possessions among previous studies mainly come from African countries [10-14,18-25] ranged from 4% to about 80%. Correspondingly, reported use during the preceding night by vulnerable groups in these regions was between 1 to 75% for any net. Hence, the results of the present study in line with some of the previous studies are too far from expected coverage of Abuja (60%) [26] and RBM (80%) [7] announcements. However, some countries including Ethiopia [23], Tanzania [25], Zambia [14], Rwanda [11], Eritrea [21], Liberia [13] and Djibouti [10] have closed or exceeded the Abuja targets. Importantly, the proportion of children and pregnant women who slept under a net during the night before the survey was considerably lower than the proportion of households that possess a net in the current study. This discrepancy between possession and utilization are consistent with the available evidence from other countries [18] and could be a matter of concern as well. The relationship between ownership and use of net might be possibly associated with the distribution of children and women of reproductive age among households owning nets. For example, among extended family owning one net, each individual may be less likely to sue a net because there are not enough nets available for everyone in the households. Unquestionably, increase access and utilization of mosquito net require assuring that all households have adequate numbers of bed net to be used by all household members, particularly children and pregnant women. Several epidemiological studies [24,25,27] have demonstrated that free or voucher scheme of insecticide-treated nets may well contribute to the household net ownership and its utilization. The experiences come from these studies might be applicable for Iran, too.

The current study also suggests that one out of every ten households reached by indoor residual spraying in the year preceding the survey. These findings are consistent with data from Angola [12], Ethiopia [23] and Djibouti [10], but considerably lower than those reported form Zambia (40%) [14] and Mozambique (52.4%) [24]. The RBM declaration [7] includes a target that 80% households in areas at risk from malaria should be sprayed with insecticide. Therefore, the IRS coverage in Iran is much lower than the level required for controlling effectively the malaria vector.

Additionally, the present study indicates that knowledge of interviewees concerning malaria symptoms and transmission are relatively modest, but mosquito net as an effective prevention method against malaria was known by an unacceptably small proportion of participants (one-third). These results are roughly similar to the existing data from the prior National surveys [10-13,18-25] with an exception for Zambia [14] where more than 80% of women reported that use of mosquito nets could prevent malaria. This lower awareness of inhabitants about preventive measures of malaria might explain some of the discrepancy between possession and use of net by vulnerable groups. Therefore, the survey suggests developing, and implementing effective health promotion policies to increase the awareness of households about the symptoms, transmission route and in particular control measures of malaria. Strong and sustainable interventions efforts including information, education, and communication activities not only maximize mosquito net use, but also improve health-seeking behaviors at community levels. Awareness of families possibly will lead them to react properly when having fever and shivering as well. More importantly, emphasis on girl's education may be probably effective to address lack of satisfactory knowledge about malaria over the long term.

Data also suggests variation by region and residency. In this study, residents in Sistan & Baluchestan province showed better knowledge and higher rate of mosquito-net use compared with other provinces. Likewise, vulnerable groups in rural areas were more likely to have or use a net compared to the urban areas. This discrepancy might be attributed to higher experience rate of malaria cases and exposures and accordingly more sensitized to malaria problem.

There are several limitations that warrant interpretation of the results. The use of mosquito net by children under-five and pregnant women was analysed only for the night prior the survey and may, therefore, not reflect the long-term pattern of net usage. Self-reported use of mosquito net as well as indoor residual spraying may also introduced bias. However, large number of included clusters, combined with well-trained local interviewers and extensive supervision, minimized these effects. Furthermore, this is the first comprehensive survey of this highly malarious region in Iran.

## Conclusions

In conclusion, massive and substantial investments are needed to coordinate national malaria control programmes towards achieving determined goals and targets by World Health Organization. Primarily, free delivery of bed net particularly, insecticide-treated bed nets, coupled with intensive nationwide education campaign may play a crucial role to achieve high ownership levels and use of net. Secondly, substantial behaviour change interventions are essential to promote malaria knowledge among individuals.

## Competing interests

The authors declare that they have no competing interests.

## Authors' contributions

The overall implementation of the first malaria indicator survey in Iran including survey design, drafting the questionnaires, data management and analysis, report writing and manuscript preparation were the results of joint efforts by multiple individuals who are listed as co-authors of this paper. They have made extensive contribution into the review and finalization of this manuscript. All authors read and approved the final manuscript.
